# Changing insurance company claims handling processes improves some outcomes for people injured in road traffic crashes

**DOI:** 10.1186/1471-2458-12-36

**Published:** 2012-01-16

**Authors:** Frederieke Schaafsma, Annelies De Wolf, Areen Kayaian, Ian D Cameron

**Affiliations:** 1 Rehabilitation Studies Unit, University of Sydney, PO Box 6, Ryde, NSW 1680, Australia

**Keywords:** Road traffic injuries, Claims handling, Rehabilitation, Health status, Return to work

## Abstract

**Background:**

Regaining good health and returning to work are important for people injured in road traffic crashes and for society. The handling of claims by insurance companies may play an important role in the rate at which health recovers and return to work is actually attained.

**Methods:**

A novel approach towards claims handling for people injured in road traffic accidents was compared to the standard approach. The setting was a large insurance company (NRMA Insurance) in the state of New South Wales, Australia. The new approach involved communicating effectively with injured people, early intervention, screening for adverse prognostic factors and focusing on early return to work and usual activities. Demographic and injury data, health outcomes, return to work and usual activities were collected at baseline and 7 months post-injury.

**Results:**

Significant differences were found 7 months post-injury on 'caseness' of depression (*p *= 0.04), perceived health limitation on activities (*p *= 0.03), and self-reported return to usual activities (*p *= 0.01) with the intervention group scoring better. Baseline general health was a significant predictor for general health at 7 months (OR 11.6, 95% CI 2.7-49.4) and for return to usual activities (OR 4.6, 95% CI 2.3-9.3).

**Conclusion:**

We found a few positive effects on health from a new claims handling method by a large insurance company. It may be most effective to target people who report low general health and low expectations for their health recovery when they file their claim.

## Background

Injuries due to road traffic crashes happen often and have a major impact on the individual and on society [1].

The effect of financial compensation on health recovery and return to work for people injured in traffic accidents has been studied extensively over the last 10 years [2-4]. Not only financial compensation but also the approach of claims handling by an insurance system towards injured people may have effect on health recovery and return to work. Recently, Casey et al. concluded that the claims management process could be improved by the inclusion of health outcome information at claim notification which would assist in identifying those at risk of delayed recovery [5]. Clear communication, professional assistance besides quick estimation of the severity and prognosis of the injury may also help speed up the health recovery and limit costs for insurance companies and health care systems. Insurance companies should provide financial assistance as well as health care assistance for best results and help the injured person to find the best treatment. They require a regulatory framework to assist this.

In New South Wales Australia compensation under the third party insurance scheme is available where people are killed or injured as a result of a motor vehicle accident. This insurance is compulsory and is regardless of the financial means of the owner or driver of the motor vehicle under the third party insurance scheme [6]. Compensation can be for economic loss (lost wages and past and future economic loss), non-economic loss and medical, rehabilitation costs. Non-economic loss is for pain and suffering and loss of quality of life.

NRMA Insurance is one of the larger insurance companies in Australia providing compulsory third party insurance for car owners. This company developed the Health Recovery Consultant (HRC) model of handling claims and Accident Notification Form (ANF) lodgements in response to proposed changes to the NSW Motor Accident Authority (MAA) which is the government regulator of motor accident insurers, with new guidelines that marked a shift in the focus from a set of minimum standards to a focus on continuous quality improvement [7]. NRMA Insurance's existing claims management process involved management of claims by injury claims consultants. It was expected that injured people who had their claims managed by one of the two new HRC models would have better outcomes in terms of their health and return to work status, a greater satisfaction with the claims management process and lower claim costs. In this study we describe the results regarding health and work status outcomes for the different HRC models and analyse potential individual predictors for better health recovery and return to work 7 months post-injury.

## Methods

### Participants

All eligible persons injured in a motor accident who filed an ANF with the insurer (NRMA Insurance) within 28 days after the motor accident between 22nd of July 2009 and 15th of February 2010 were included. Injured persons were excluded from the study if they:

• had a catastrophic injury claim

• were less than 18 years of age

• lodged an ANF involving a pure Workers Compensation Claim

• lodged an ANF involving an inward sharing claim

• lodged an ANF involving compensation to relatives (deceased)

• were represented by a lawyer within 2 weeks of date of ANF lodgement

• lodged a Personal Injury (PI) claim within 2 weeks of date of ANF lodgement

• lodged an ANF late (more than 30 days from injury)

• the claim was finalised within 2 weeks of date of ANF lodgement

Eligibility of the injured person to submit a claim was evaluated as soon as the claim was lodged with the insurer. Injured persons who met the inclusion criteria and who agreed to participate were assigned to one of the three teams based on the capacity of the teams to handle another claim. As this project was an evaluation of a quality improvement activity within one insurance company, ethics committee approval was not considered necessary.

### Intervention

The intervention differed from the usual claims management process by "providing an early intervention service (specific and time focussed), risk screening of ANFs, compliance with evidence based management, facilitating and arranging general practitioner conferencing with the injured person and the recovery consultant, psychological screening at an early stage of the claim (less than 6 weeks after lodgement), facilitating early return to work, following the consistent communication protocol (ie communicating clearly and directly with the injured person and acknowledging their ongoing issues eg re pain), proactively resolving disputes and prompt approval of treatments that were reasonable and necessary. These aspects of the intervention were described in protocols and a training program was developed for the protocols. The consultants in the intervention group underwent ongoing education for the duration of the trial and the consultants were monitored for compliance. Claims in the intervention group were managed by either a consultant trained in injury management, or by a consultant with an allied health background trained in claims management. These consultants could spend approximately 50% more time on each claim compared to the consultants in the control group.

The control group consisted of injured persons who had their claims managed by the existing processes of the insurance company. These claims were processed by injury claims consultants. The consultants in the control group were not informed regarding the protocols, they did not receive the same initial or ongoing training and they were not monitored or given feedback according to their adherence to those protocols.

### Materials and data collection

One month after the injury (baseline) demographic, pre-injury health, and injury data, health outcomes, return to work and usual activities, and economic data were collected using a survey questionnaire. Participants were offered the choice to complete the survey by phone, mail, or email. For pre-injury health we asked the participant to rate their general health prior to the accident (excellent, very good, good, fair, and poor). For the injury data we used the level of pain (scores between 0 and 10), the number of injuries, and the Modified Abbreviated Injury Scale (MAIS) score from NRMA Insurance. The MAIS scores between 1 and 6 where score 1 is used for minor injuries such as skin injuries, joint strains/soft tissue injuries and score 6 is only used when there is indication that the claimant has a catastrophic or an untreatable injury. See Additional File 1.

Health outcomes included the Hospital Anxiety and Depression Scale (HADS) and the Medical Outcomes Survey Short Form-12 (SF-12). The HADS is a brief self-report questionnaire (14 items), which assesses anxiety (HADS-A) and depression (HADS-D) as two distinct dimensions in non-psychiatric populations [8,9]. Seven items relate to each dimension, requiring answers on a 4-point scale (e.g. from 0 'not at all' to 3 'very often indeed'). It has been used widely in clinical settings where anxiety and depression can co-occur with physical pathology [10]. A cut-off score of 8+ is used to screen for 'caseness'. Raw scores for each domain can be interpreted as follows: between 8 and 10 (mild cases), 11-15 (moderate cases), and 16+ (severe cases) based on population norms from the UK published by Crawford et al. [11]. HADS has demonstrated good internal consistency with Cronbach alpha values from .68 to .93 for HADS-A, and from .67 to .90 for HADS-D [8].

The SF-12 is a health survey constructed to monitor general health, both physical and mental health, in general populations [12]. The SF-12 has 12 questions selected from the SF-36 Health Survey [13]. Scoring of the SF-12 provides results on 8 domains (physical functioning, role physical, bodily pain, general health, vitality, social functioning, role emotional, and mental health). Two component scores, physical and mental component summary, are derived from the domain scores, the domain scores and component scores are standardised to a mean of 50 and standard deviation of 10.

Return to work outcomes included "If you were employed before the accident, have you returned to work?" with answer options "yes full duties, yes modified duties, no incapacitated for work, no seeking work, no retired, no not working not seeking". Usual activities question included "Regardless of whether you were employed before the accident, have you returned to your usual activities?" with answer option "yes, no". This question was followed with "On a scale 100% (fully returned to usual activities) to 0% (not at all returned to usual activities) - in your opinion where do you feel you are in your recovery process to usual activities?"

All participants who completed the 1 month survey were invited to complete a follow-up survey at 7 months from the date of injury. Based on 5 points on SF-12 being smallest between-group difference worth detecting, it was estimated that 60 participants in each team would provide sufficient power (β = 80), with α = 0.05, and allowing for 30% dropouts.

### Statistical analysis

Baseline characteristics of participants were analysed using *T*-test for two independent groups for normally distributed data and the Mann-Whitney U tests for data that was not normally distributed. F-tests or Chi-square tests were performed for categorical data. Categorical data were recorded into dichotomous variables prior to the regression analysis. GLM repeated measures analyses were carried out to assess the change from baseline (1 month post injury) to 7 months post injury for both groups, and to assess whether the change in outcome over the course of 6 months was influenced by team assignment. Univariate analysis was carried out using simple logistic regression to evaluate factors significantly associated with general health and return to usual activities at 7 months. The significant factors were included in a backward conditional logistic regression analysis as predictor variables. We performed all statistical analysis using SPSS version 16.0.

## Results

**Figure 1:**
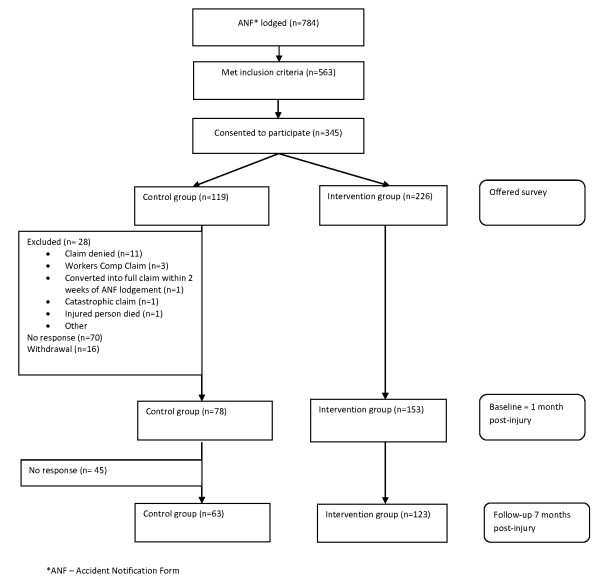
**Flow chart**.

Figure 1 presents the recruitment flow for the HRC evaluation. A total of 784 ANFs were lodged with NRMA Insurance of which 345 claims met the initial inclusion criteria and agreed to participate in the evaluation. Another 28 participants were excluded for various reasons after the allocation to the two groups (see Figure 1). Of the 317 injured people 231 injured people completed the initial survey (73% participation rate) and at 7 months post injury data were obtained from 186 participants. The analyses were carried out on these 186 participants. There was no difference in rate of follow up between the two groups. Participants who dropped out during the trial were younger compared to those who were followed up at 7 months (38 years (SD = 15) versus 45 years (SD = 17), *p *= 0.01). Twice as many females dropped out at 7 months compared to males (23% versus 13%, F- test *p *= 0.08). Twice as many participants who were employed (either full time or part time) prior to the accident dropped out at 7 months compared to participants not employed prior to injury (n = 7, 12%, F-test *p *= 0.06). There was no difference in pre-injury health and reported general health 1 month after injury between those who dropped out and those who were followed up at 7 months.

Demographic and social characteristics of participants in the two groups are shown in Table 1. Overall there were no differences in baseline characteristics between the two groups.

**Table 1:** Participant Characteristics at baseline

	Total (n = 186)	Control group (n = 63)	Intervention group (n = 123)	Test statistic
Age mean (SD)		46.5 (18.7)	44.2 (15.8)	T-test, *p *= 0.38

Male gender n (%)	63 (34)	21 (33)	42 (34)	F-test *p *= 1.00

BMI		26.8 (5.9)	25.0 (4.0)	T-test, *p *= 0.05

Smoking n (%)	14 (7)	4 (6)	10 (8)	F-test, *p *= 0.78

Pre-injury general health being very good or excellent n (%)	147 (79)	47 (75)	100 (81)	F-test, *p *= 0.34

**Educational Level n (%)**				X^2^, *p *= 0.31

Postgraduate degree/graduate diploma/bachelor degree	60 (32)	18 (29)	42(34)	

Advanced diploma/certificate	63 (33)	19 (30)	44 (36)	

Primary/Secondary education	63(33)	26 (41)	37 (30)	

Pre-injury employment Status n (%)*				F-test, *p *= 0.61

Employed	130 (70)	42 (68)	88 (72)	

Unemployed	54 (30)	20 (33)	34 (28)	

**Injury Category n (%)**				X^2 ^*p *= 0.52

WAD	108 (58)	35 (56)	73 (60)	

STI multiple	23(12)	8 (13)	15 (12)	

STI single	12 (7)	5 (8)	7 (6)	

Orthopaedic	20(10)	6 (10)	14 (11)	

Joints	9(5)	4 (6)	5 (5)	

Other	13 (9)	5 (9)	8 (7)	

**Number of Injuries mean (SD)**	2.5 (1.8)	2.6 (1.7)	2.5(1.9)	T-test *p *= 0.86

**Injury severity MAIS mean (SD)**	1.8 (2.4)	2.1 (2.7	1.7 (2.2)	T-test *p*= 0.39

*STI *soft tissue injury, *WAD *whiplash associated disorder

*Data missing for two participants

Seventy percent of participants were employed pre-injury of which the vast majority were white collar workers. Five percent of these workers were employed in modified duties prior to the injury. This means that these workers performed duties which were modified from the usual job description to accommodate the employee and there could be reduction in hours, other than normal duties, or exclusion of some duties.

Fifty five percent of injuries were whiplash associated disorders (WAD), followed by multiple soft tissue injuries (12.7%). The two groups were similar with regard to injury category, the number of injuries sustained, and severity of injury using the MAIS system. See Table 1.

At baseline the self-rated average percentage recovery was 58% (SD = 27%), which increased to 78% (SD = 24%) at 7 months follow-up with no difference between the two groups (*T*-test, *p *= 0.41). The majority (87%) reported that pain was present at baseline and the average pain level was 4.5 (SD ± 2.6). At 7 months 57% reported pain with average pain level of 2.7 (SD ± 2.8) with no difference between the two groups (*T*-test, *p *= 0.92).

### Quality of life

Table 2 shows SF-12 norm-based scores for all participants at baseline compared to the Australian general population which has a mean score of 50 and standard deviation of 10. Both groups improved on health scores between baseline and 7 months post injury. We found that there was a significant difference for the domain of perceived health limitation for physical activities between the two groups with the intervention group improving more than the control group over time (*p *= .033).

**Table 2:** SF-12 and HADS scores Mean (SD) health outcomes and change from baseline to 7 months post injury

	Baseline (1 month post injury)		Change from baseline to 7 month post injury		Time × team (p)
**SF-12**	**Control group (n = 63)**	**Intervention group (n = 123)** **(n = 123)**	**Control group (n = 63)**	**Intervention group (n = 123)**	

Physical functioning	35.3 (11.1)	38.3 (11.2)	8.3 (11.1)	9.2 (11.1)	.601

Role physical	37.5 (15.2)	36.8 (9.8)	9.3 (10.8)	12.8 (10.5)	.033

Bodily pain	35.8 (12.1)	35.4 (11.8)	9.2 (12.1)	12.7 (11.5)	.058

General health	43.9 (12.5)	45.9 (12.3)	1.1 (11.7)	3.5 (12.3)	.199

Vitality	43.3 (11.5)	43.6 (11.7)	6.1 (10.9)	6.5 (14.4)	.849

Social functioning	41.4 (13.7)	39.9 (13.7)	5.4 (13.9)	8.8 (14.3)	.130

Role emotional	40.6 (13.5)	41.2 (13.3)	6.1 (14.5)	8.1 (13.4)	.348

Mental health	43.5 (11.8)	44.0 (11.6)	5.9 (14.0)	7.4 (13.7)	.489

Physical component score	36.5 (10.8)	37.7 (10.8)	7.6 (9.7)	10.2 (10.9)	.113

Mental component score	45.3 (7.7)	44.9 (12.6)	4.4 (14.7)	6.0 (13.7)	.473

**HADS**					

Anxiety symptoms	7.9 (4.7)	7.2 (4.4)	1.7 (4.4)	1.9 (4.3)	.682

Depressive symptoms	5.9 (4.7)	5.8 (4.5)	1.4 (4.0)	2.5 (4.2)	.568

Abbreviations: *SF-12 *Medical Outcomes Survey short form - 12, *HADS *Hospital Anxiety and Depression Scale, *SD *standard deviation

Note: all values are Mean (SD)

### Anxiety and depressive symptoms

At baseline, anxiety and depression symptoms were prevalent across both teams (Table 3). The number of participants who screened positive for anxiety or depression was reduced by 14% by 7 months post injury. This resulted in 31% of participants screening positive for anxiety and 19% for depression. In terms of 'caseness' (any participant with a score greater than 8), a marginally significant difference was found between the teams at 7 months post injury with regard to depression (*p *= 0.044), with the intervention group having a lower number of participants reporting depression compared to the control group.

**Table 3:** Number and percentage of participants with mild, moderate, and severe anxiety and depression at baseline and 7 months post injury

	Baseline (1 month post-injury			7 months post-injury		
	**Control group (n = 62)**	**Intervention group (n = 123)**	Statistic test* *p*-value	**Control group (n = 63)**	**Intervention group (n = 123)**	Statistic text * *p*-value

Anxiety categories:			.636			.415

Mild (8+) n (%)	10 (16)	22 (18)		12 (19)	13 (11)	

Moderate (11-15) n (%)	15 (25)	25 (20)		8 (13)	18 (15)	

Severe (16+) (%) n (%)	4 (7)	5 (4)		3 (5)	3 (2)	

'Caseness' (≥ 8)	38 (50)	52 (42)	.642	23 (37)	34 (28)	.241

Depression categories:			1.000			1.000

Mild (8+) n (%)	10 (16)	18 (15)		10 (16)	11 (9)	

Moderate (11-15) n (%)	8 (13)	15 (12)		6 (10)	5 (4)	

Severe (16+) n (%)	3 (5)	5 (4)		1 (2)	1 (1)	

'Caseness' (≥ 8)	25 (31)	38 (31)	.742	17 (28)	17 (14)	.044

*X^2 ^or F-test

### Return to work and usual activities

Eighty two percent of participants who were employed pre-injury (n = 130), had already returned to work at baseline, either in full capacity or modified duties (Table 4). At 7 months there were only seven participants (5%) who were still incapacitated. The two groups were similar in their return to work rate. There were a significantly higher number of participants in the control group who reported they had not yet returned to their usual activities at 7 months, compared to the intervention group (F-test, *p *= 0.005).

**Table 4:** Employment status, return to work and return to usual activities

	Total (n = 186) N (%)	Control group (n = 63) n (%)	Intervention group (n = 123) n (%)	Statistic test* p-value
Employed pre-injury	130 (70)	42 (67)	88 (72)	0.61
• Full duties	123 (95)	38 (91)	85 (97)	0.32
• Modified duties	7 (5)	4 (9)	3 (3)	

Employed 1 month post-injury	106 (57)	34 (54)	72 (59)	0.64
• Full duties	64 (60)	17 (50)	47 (65)	0.14
• Modified duties	42 (40)	17(50)	25 (35)	

Returned to usual activities at 1 month post-injury	59 (32)	23 (37)	36 (29)	0.32

Employed 7 months post-injury	126 (68)	39 (62)	87 (71)	0.45
• Full duties	100 (79)	27 (69)	73 (84)	0.09
• Modified duties	26 (21)	12 (31)	14 (16)	

Returned to usual activities at 7 months post-injury	137 (74)	38 (60)	99 (81)	0.01

*X^2 ^or F-test

### Type of claims

The 186 ANFs were converted to a personal injury claim in 56 cases, and more frequently in the control group (41% versus 25%; F-test, *p *= 0.04). Forty three personal injury claims included an economic loss component with no difference between the two groups (F-test, *p *= 0.14). Twenty one percent of the ANFs became legally represented with no difference between the two groups (F-test, *p *= 0.19). For finalised claims, all except two cases were ANF claims. For open claims the majority (86%) of claims were converted to personal injury claims. Legal representation was correlated to injury severity as measured with the MAIS score from NRMA Insurance (Pearson 0.21, *p *< 0.001), number of injuries (Pearson 0.44, *p *< 0.001), level of pain at baseline (Pearson 0.27, *p *< 0.001), and the level of satisfaction with NRMA Insurance (Pearson -0.18, *p *= 0.01). The more severe the injury, the more injuries, the higher level of pain and the lower level of satisfaction were related to a higher chance of participants with legal representation.

### Predictors of health and return to usual activities

Because almost all participants had returned to work no further analyses related to work were performed. Table 5 shows the variables that were associated with return to usual activities at 7 months. Demographic (age, gender, marital status) and socio-economic factors (level of education, occupational group, pre-injury employment status or satisfaction with employment) were not found to be associated with return to usual activities.

**Table 5:** Univariate analysis of predictors for return to usual activities at 7 months post-injury

Variable	B	SE	OR	CI
Good baseline general health	1.53	0.36	4.61	2.28-9.29

Higher level of pain	-0.30	0.08	0.74	0.64-0.86

Economic loss claim Yes	-1.50	0.56	0.22	0.08-0.66

Higher baseline HADS depression score	-0.16	0.04	0.86	0.80-0.92

Expected longer duration for return to usual activities	-1.07	0.24	0.34	0.21-0.56

Stepwise backward conditional logistic regression analysis with return to usual activities at 7 months as dependent variable showed that expected longer duration of return to usual activities (OR 0.42, 95% CI 0.26-0.68), good baseline general health (OR 2.60, 95% CI 1.05-6.45), and having a claim including an economic loss component (OR 0.22, 95% CI 0.06-0.84) explained 39.4% of the variation.

Table 6 shows the variables that were associated with good general health at 7 months (measured with the first question of the SF-12). No association was found for injury severity measured with the MAIS, BMI, smoking or having pain at baseline.

**Table 6:** Univariate analysis of predictors for good or excellent general health at 7 months post-injury

Variable	B	SE	OR	CI
Good pre-injury general health	2.45	0.74	11.62	2.73-49.37

Good baseline general health	1.99	0.42	7.28	3.18-16.64

WAD	0.85	0.40	2.34	1.07-5.13

Higher Age	-0.04	0.01	0.96	0.94-0.98

Economic loss claim Yes	1.69	0.75	0.18	0.04-0.80

Higher baseline HADS anxiety score	-0.16	0.04	0.85	0.78-0.93

Higher baseline HADS depression score	-0.20	0.04	0.83	0.76-0.91

Baseline PCS score	0.17	0.03	1.09	1.05-1.14

Baseline MCS score	0.06	0.01	1.06	1.02-1.09

Stepwise backward conditional logistic regression analysis with good or excellent general health at 7 months (measured with the first question of the SF-12) as dependent variable showed that baseline general health (OR 4.01, 95% CI 1.57-10.25), together with age (OR 0.96, 95% CI 0.93-0.98), and HADs depression scores (OR 0.86, 95% CI 0.78-0.95) remained significant predictors for general health at 7 months and could predict 84.9% of the cases.

## Discussion

This study has evaluated the effectiveness of the existing claims management process compared to a recently introduced Health Recovery Consultant model for the largest general insurance company in Australia (NRMA Insurance). It was demonstrated that it was feasible to engage injured people in an evaluation and to achieve a good level of follow-up (81%) at 7 months after injury. The groups were well matched at baseline, with no difference in injuries sustained. After 7 months we measured a difference between the two groups for 'caseness' of depression, perceived health limitations for activities (measured with the SF-12), and actual return to usual activities with the intervention group scoring significantly better. Almost all participants had returned to work at 7 months.

These positive results for the intervention group may be a result of more personal attention, easy communications lines, and advice from the health consultants of the insurance company that are based on evidence based medicine resulting in a more efficient use of medical health services.

The evaluation also identified useful predictors of injury outcome that could be used to further develop the Health Recovery Consultant models. The first question of the SF12 ("In general, would you say your health is: excellent, very good, good, fair, or poor?") at baseline is a good predictor of health status at 7 months and return to usual activities. This supports the use of this question as a screening tool at the first contact with the injured person. The number of injuries and the severity of the injury measured with the MAIS and pain reported at baseline are correlated to legal representation and could also be used to decide about the intensity of claim management by the insurance company to respond to increased likelihood of legal representation.

In occupational health studies, prediction of return to work has been a major topic for research studies [14-17]. It was recognized that return to work programs focussing particularly on injured people with low expectations of health recovery and return to work has significant positive effects and improves health and return to work outcomes. In this study the majority of participants (95%) had returned to work at 7 months but still reported health problems such as depression (19%) and anxiety (31%). Based on the population norms from the report by Crawford et al. [11] our results indicate that by 7 months most claimants returned to present UK norm in terms of anxiety. In terms of depression, only the intervention group had reached a percentage of 'caseness' comparable to the normal UK population. This shows not only the major impact a motor vehicle accident can have on a person's life but also that the claims handling method by the insurance company may influence health outcome such as depression to a small extent. The long term negative effect on health is in line with literature that reports increasing evidence that a substantial proportion of people injured in road traffic crashes who attend hospital emergency departments with only minor injuries have continuing physical symptoms, major psychiatric consequences, and effects on everyday life at 1 year [18,19]. Post-traumatic stress disorder, phobic travel anxiety and limitation can persist for even longer periods [20]. Persistent medical and financial problems are chronic stressors, which pose demands on the individual's coping abilities and may thus make it more difficult for them to overcome the psychological consequences of the trauma. It is also likely that they are continuing reminders of the trauma, which may make it more difficult for the individual to see the accident as something from the past. Some of these people will still need psychological or medical assistance after 7 months and it is therefore important for insurance companies to be able to screen for this risk at an early stage.

Results of this study are somewhat different from the findings by Buitenhuis et al. who found that age and concentration complaints 1 month after a traffic accident, for individuals with neck complaints, are independent predictors of work disability after 1 year [21]. In our study we found that low baseline general health, the expected longer duration of return to usual activities, and having filed an economic loss claim negatively influenced return to usual activities. But the vast majority of participants (95%) who were working prior to the injury had returned to work, including those with neck complaints (n = 108) after 7 months, opposed to only 59% in the study by Buitenhuis et al. This difference in return to work rate may be explained by different social security systems or by less overall impairment for the participants in our study. Baseline health data of injured persons in the HRC evaluation were similar to those found in the Accident Care Evaluation trial (ACE) [18]. This is an Emergency Department inception cohort study carried out in the Australian Capital Territory, investigating health outcomes in people sustaining musculoskeletal injuries in road traffic accidents. An exception is the Mental Component Score of the SF12 which was less impaired in this evaluation. This is most likely to be due to the different population groups from which the participants were recruited. All participants in the ACE trial were recruited from the Emergency Department while participants in the HRC evaluation were recruited from a pool of ANF lodgements with accidents not always resulting in a significant injury [18].

From the participants who dropped out, we found that the majority were women, were younger and were working (full time or part time) prior to the accident. It is understandable that people who are working have less time to spend to continue participate in the trial. Why more women dropped out and why they tended to be younger may need further exploration in future studies.

A weakness of this study is the lack of information on compliance to the actual implementation of the intervention. We have no exact data on how many extra personal contact there was between the consultant from the insurance company and the participant, or to what extent the recommendations were according to evidence based guidelines. However, the better health related outcomes at 7 months may be a result from the larger investment of the consultants to respond to the needs of the claimant. Future studies need to improve documentation on compliance or implementation of the intervention. It may be feasible to further fine tune the Health Recovery Consultant model to allocate greater case management resources to injured people who are at greater risk of poor recovery based on factors evident soon after injury such as high levels of pain, a high number of injuries, or poor physical or mental health status at baseline. This may form a rationale for the NMRA Insurance to implement the Health Recovery Consultant models particularly for these groups. Another weakness is that there was no randomization of the participants. Although, baseline characteristics showed no significant difference on injury severity and type of injury, it is recommended that future studies should randomize participants.

Further research is needed to further understand which aspects of the new model have resulted in this positive effect. Baseline health indicators may be important predictors for health status at 7 months post-injury and could be used as indicator for extra input by the insurance company. It was suggested that the association of greater severity of injury with legal representation may relate to additional factors such as the person's concern about their health status.

## Conclusion

We found a few positive effects on health from a new claims handling method by a large insurance company. It may be most effective to target people who report low general health and low expectations for their health recovery when they file their claim.

## Competing interests

The authors declare that they have no competing interests.

## Authors' contributions

IC conceived the study, participated in its design, implementation and analysis and had overall scientific responsibility. AdW and AK were involved in the study design and also coordinated the study implementation, recruitment and data collection. FS and AdW coordinated the statistical analysis and interpretation of the results. FS wrote the first draft of the manuscript. All authors helped to refine the manuscript, read and approved the final version.

## Pre-publication history

The pre-publication history for this paper can be accessed here:

http://www.biomedcentral.com/1471-2458/12/36/prepub

## Supplementary Material

Additional file 1 **The Modified Abbreviated Injury Scale (MAIS) Classification**. An explanation of each MAIS score. Click here for file
