# Supplementary material for: Changing insurance company claims handling processes improves some outcomes for people injured in road traffic crashes
Source: BMC Public Health. 2012 Jan 16;12:36. doi: 10.1186/1471-2458-12-36 (PMC3292930; doi:10.1186/1471-2458-12-36)
Supplement: Additional file 1 — The Modified Abbreviated Injury Scale (MAIS) Classification. An explanation of each MAIS score. [file 1471-2458-12-36-S1.DOC]

Additional File 1

The Modified Abbreviated Injury Scale (MAIS) classification

| MAIS Score | MAIS explanation |
| --- | --- |
| 1 | Use for claimants with minor injuries such as skin injuries, joint strains/STIs, unilateral ear and eye injuries (unless involving retinal detachment or sclera injury), minor penetrating injury to the skin, closed mandible fractures and fractures of fingers and toes. |
| 2 | Use for claimants with moderate injuries including mild concussive injuries, muscle tendon & ligament injuries, closed fractures of long bones (except femur), dislocations, stable pelvic fractures, disc injuries, internal injuries (typically OIS* 1, II) and partial thickness burns. |
| 3 | Use for claimants with serious injuries including small brain injuries of the cerebrum, moderate concussive injuries, spinal injuries (with transient neurological impairment or radiculopathy), femur fractures and open fractures of the humerus, radial, ulnar, tibial, fibular, unstable pelvic fractures, internal injuries (typically OIS III), full thickness burns, major skin injuries with blood loss, vascular injuries of face, upper and lower extremities. |
| 4 | Use for claimants with severe brain injuries including small cerebellum injuries, large cerebral injuries, DAI† 6-24 hrs, spinal cord contusions, internal injuries (typically OIS IV) and vascular ‘lacerations’ of head, neck, thorax and abdomen. |
| 5 | Use for claimants with critical injuries including major cerebellum brain injuries, brain stem compression, bleeding or infarction, DAI >24hrs, spinal cord injury C4 or below, internal injuries with life threatening vascular compromise (typically OIS V), vascular transection or bilateral laceration. |
| 6 | Do not use unless there is indication that the claimant has an untreatable injury in the brain (i.e. brain stem laceration), a complete or bilateral transection of major vessel or the heart, a cervical spinal cord injury C3 above or a catastrophic whole body injury (e.g. crushed chest). |
|  | Not to be used for claimants who die immediately as a result of the accident – i.e. compensation to relatives claim |
| 9 | Use in cases where no medical information is available, a person dies as a result of the MVA, and for claimants who have medical complications or psychological illness reported as consequence of the accident but no physical injuries. |

* OIS – Organ Injury Scale

† DAI – Diffuse Axonal Injury
